# Blooming markets: The economic dynamics of the Nepal rose trade

**DOI:** 10.1016/j.heliyon.2024.e35585

**Published:** 2024-08-02

**Authors:** Prakash Awasthi, Shiva Prasad Adhikari

**Affiliations:** Institute of Agriculture and Animal Science, Tribhuvan University, Nepal

**Keywords:** *Cut flower***,***demand*, *Export*, *Import*, *TEPC*

## Abstract

Thanks to its excellent climate and varied agroecological zones, Nepal has been a significant player in the global rose market. The geographical variety of the nation makes it possible to grow roses all year long, making it a desirable location for rose export and cultivation. The production and export of roses have significantly increased in recent years. The rose trade has increased in Nepal for several reasons. Investment in the industry has been stimulated by government measures to support floriculture and offer incentives to rose farmers. Additionally, the effective distribution of roses to domestic and foreign markets has been made possible by better transportation infrastructure. The growth of the global market has greatly aided the development of Nepal's rose industry. The growth of rose cultivation in Nepal has significantly impacted the economy and employment. It is a good source of revenue through internal sales, export, and other activities that provide value addition. The sector creates direct farm jobs while local communities benefit from indirect employment in supporting industries. In addition, there is increased job opportunities due to seasonal spikes in demand. Rose exports have expanded due to a rise in demand for Nepali roses, particularly in European markets and nearby nations like India. The favorable trade agreements Nepal has with several nations have helped to increase its exports even more. But issues still exist in the rose trade sector. Since maintaining consistent quality is necessary to compete in the international market, quality control and standardization continue to be areas of concern. The rose business in Nepal is also vulnerable to changes in global demand and competition from other nations that grow roses. Further expansion of the industry will help it overcome problems like limited technology and training hence leading to its fast development; this calls for government support as well as market expansion. In conclusion, the rose trade environment in Nepal is a fluid and changing one. Due to favorable circumstances, government assistance, and global demand, the sector has seen significant expansion. Continuous efforts in quality control, market diversification, and innovation are necessary to maintain and grow this sector. The rose trade in Nepal has the potential to significantly boost the nation's agricultural exports while promoting economic growth in rural areas.

## 1 Introduction

Part of the Rosaceae family, roses *(Rosa* spp.*)* are among the most beloved and iconic flowers in the world. Termed as queen of flowers in the world, it is familiar with different names such as ‘Gulaf in Nepal, Gulab in India, Méiguī in China, Roza in Bulgaria, Roos in the Netherlands, Rozu in Japan, etc.’ [1]. With the ability to grow up to 6 m, plants possess a wide range of origins, the vast majority of species are minor populations that are native to Asia and also have a good amount of traces to continents like Africa, Europe, and North America [2]. Flowers are a vital part of human society starting from joy to sorrow among them rose has gained a vast popularity. Roses from decades captured the human heart because of their exquisite beauty, fragrant aroma, and rich symbolism and are listed among the most demanded cut flowers in the world [3]. In addition to their aesthetic appeal, roses are treasured for the wide range of emotions and thoughts they may represent. They have been used in literature, the arts, and other types of culture for a very long time because they are connected to love, passion, and romance. Roses have deeper implications beyond only matters of the heart, such as purity, companionship, and memory. It is a woody perennial shrub with more than 100 different kinds and countless varieties. It is present in every range, different hues, sizes, and scents, and is the floral monarch. These flowers are frequently used in bouquets, fragrances, cosmetics, and food products like rosewater and teas with rose flavoring. The present-day cultivars are hybrids that can have many classifications like Floribunda, Hybrid Tea, Ramblers, climbers, etc. This guide is used mostly for production systems and management of cultivated hybrid tea roses mostly for cut flower use. Roses thrive in a climate with moderate temperatures, lots of sunlight, and between 15 and 27 °C temperature ranges [4].

Optimal irrigation range of 3.70–5.29 mm per day Under water deficit conditions, providing only 67 % of the necessary irrigation resulted in a decreased yield of marketable rose flower stalks cultivated in three distinct substrates. Rose flowers exhibit antioxidant and antiseptic properties and are a source of vitamins A, B3, C, D, and E. Rose hips, alternatively known as Apothecary Rose, are packed with minerals, saturated and unsaturated fatty acids, carotenoids, and phenolic compounds [5]. Rose water and rose oil derived from Rosa damascena are composed of phenyl ethyl alcohol, citronellol (1.8–7.2 %), nonadecane (10.5–40.5 %), geraniol (0.9–7.0 %), and nerol (0.9–7.0 %) as primary constituents, alongside eicosane, heneicosane, tricosane, geranyl acetate, and eugenol. Petals are particularly rich in citronellol and contain abundant aromatic volatile oils, gallic acids, and quercetinic acid, as well as terpenes, glycosides, flavonoids, anthocyanins, carboxylic acids, myrcene, vitamin C, kaempferol, quercetin, and geraniol [6,7,8].

Rose flower production yielded 2,24,166 per hectare, generating a gross revenue of Rs. 3,80,242, with a benefit-cost ratio of 1.29 [9]. The minimum survival rate observed in a rose nursery within a greenhouse was 65 % while the net present worth (NPW) of the greenhouse investment amounted to Rs. 453,221, with an internal rate of return (IRR) of 53 % and a benefit-cost ratio (BCR) of 4.5 [8].

Out of 77 districts, 44 districts were involved in the floriculture business with an investment of around $70 million [10]. The great rise of the floriculture sector was also due to the involvement of more women in farming [11]. The floriculture sector in Nepal comprises 732 entrepreneurs working across 44 districts. It engages more than 43,000 individuals directly or indirectly and has attracted investments exceeding 7000 million rupees (FAN, 2021). Among floriculture sector cut flowers particularly rose gained a ranked priority because of its greater vase life, popularity among youngsters, attraction towards Western culture, and more use of rose flowers in the auspicious occasions and celebrations like marriage, holy thread ceremonies, etc. [12]. The import of fresh cut and buds of rose was around 1900 kg valuing $1692.64 [13]. In the year 2021/22, cut flowers contributed 0.06 % to Nepal's GDP, with roses being the major contributor [13]. Roses and other flowers have been sent from Nepal, mostly to nations that are close by, such as India. The export of roses from Nepal has been prompted by the demand for roses in India, notably on special events like Valentine's Day. Roses have been exported from Nepal, particularly from the Kathmandu Valley, to places like Kolkata and Delhi.

Rose farming in Nepal presents various possibilities influenced by climatic, geographic, economic, demographic, and cultural factors.

### 1.1 Climatic factors

Nepal's varied climatic regions, spanning from tropical to alpine, create optimal conditions for cultivating a wide range of rose varieties. The diverse altitudes and climates allow for year-round rose cultivation, facilitating a steady supply and consistent income. Employing advanced methods like greenhouse farming enables farmers to overcome adverse climatic impacts, thereby sustaining rose production even amidst fluctuating weather conditions.

### 1.2 Geographic factors

Nepal is divided into the Himalayan foothills, Terai plains, and mid-hills. These areas present varied terrains for cultivating diverse rose species. Their proximity to urban hubs such as Kathmandu and Pokhara facilitates convenient market access for fresh roses, cutting down on transportation expenses and ensuring higher product freshness [4]. Additionally, ample water resources from rivers and monsoon rains cater to the irrigation demands necessary for successful rose cultivation in these regions.

### 1.3 Economic factors

Increasing domestic and global demand for roses represents a promising economic opportunity. Roses are favored for weddings, festivals, and daily adornment, ensuring a consistent market. As a high-value crop, they offer substantial income compared to traditional agricultural products. Nepal's roses hold export potential to neighboring nations like India and China, promising considerable economic advantages. Furthermore, rose farming has the potential to generate employment across various sectors such as planting, harvesting, processing, and marketing, thereby contributing significantly to rural development.

### 1.4 Demographic factors

The presence of a youthful and potentially adept workforce can catalyze innovation and effectiveness in rose farming methods. Given the trend of youth migrating abroad for employment, promoting rose farming offers local job prospects, potentially mitigating migration rates. Heightened education and awareness about contemporary farming techniques among young individuals can elevate productivity and standards in rose cultivation.

### 1.5 Cultural factors

Roses hold a profound significance in Nepali culture, integral to religious rituals, festivals, and social gatherings, ensuring consistent domestic demand. The longstanding tradition of floriculture in Nepal offers a solid base of expertise and wisdom for expanding rose cultivation. Valued for their beauty and cultural importance, roses enjoy widespread acceptance and appreciation, making them a preferred option for both producers and consumers alike.

### 1.6 Opportunities for rose farming in Nepal

1. Agro-Tourism: Combining rose farming with agro-tourism can attract visitors and generate additional income.
2. Organic Farming: Leveraging Nepal's natural resources to promote organic rose farming can attract premium markets and environmentally conscious consumers.
3. Value-Added Products: Developing products like rose oil, rose water, and dried rose petals can diversify income sources and add value to the basic farming operations.
4. Training and Development: Government and NGO programs focused on training farmers in advanced techniques can improve productivity and sustainability.
5. Technological Integration: Utilizing modern farming technologies like drip irrigation, greenhouse farming, and automated systems can enhance yield and quality.
6. Cooperatives and Associations: Forming cooperatives can help small farmers pool resources, share knowledge, and access better markets and financing options.

Overall, rose farming in Nepal offers significant possibilities driven by favorable climatic, geographic, economic, demographic, and cultural conditions. With proper support and development, it can become a lucrative and sustainable occupation for many Nepali farmers.

## 2 Review of literature

Rose is one of the most beautiful and most demanded cut flowers in the world. Ecuador is the leading producer, the Netherlands is the top exporter whereas the USA is the top importer of rose in the world [14]. In European countries trade of rose as well as other cut flower always remain at a peak as peoples demand these flowers for different purposes like decorations, celebrations, raw materials for industries and pharmaceuticals, etc. but comparing the trade with country like Nepal located in the south Asian region of world the trade is very less. In the present scenario the trend of adopting rose on different occasions is increasing in Nepal as the society is attracted towards Western culture. As the demand for flowers continues to climb both locally and internationally, farmers are gradually becoming more interested in the cultivation of rose flowers [15]. But when analyzing the periodic data of trade, production is quite imbalance in Nepal. The production and trade of roses in Nepal may be limited for several reasons as climatic and altitude variations, lack of technology and expertise, lack of proper transportation and infrastructure like storage facilities, dominance of seasonal flowers, inappropriate market access and facilities, limited research and development, improper grading system, improper quarantine practices and wide spread of pests and diseases, untimely extension services. Along with these problems numerous obstacles, such as logistical problems, quality control problems, and competition from other flower-producing nations like Kenya, Ethiopia, and the Netherlands, beset Nepal's rose export sector [14]. Another major problem in the production of rose is the inequality in budget allocation by the government and discrimination in running research, plantation related activities by FAN in different parts of the country.

Nepalese floriculture creates employment opportunities for more than 44,000 individuals and currently achieves an annual turnover amounting to NRs. 230,34,08,000 [16]. Among Nepalese floriculturists, only 14 % receive extension services, and 31 % have been trained in production, marketing, promotion, linkages, entrepreneurship, and leadership while propagation materials make up 80 % of the country's floriculture exports, primarily to India, while Nepal imports a significant share of live plants and cut flowers from India, approximately 38.40 % [17]. According to trade and promotion center report on 2022 Nepal predominantly exports floriculture products to the U.S.A. (38.05 %), Japan (29.38 %), and Indonesia (16.07 %). 75 % of the workforce is employed in agriculture, with women constituting the majority. Specifically, in the flower industry, women account for 64 % of the workforce, as noted by Ref. [18].

Rose farming is becoming more and more popular among farmers as domestic and international demand for flowers rises. The lack of a land leasing policy for integrated farming, difficulties obtaining a bank loan, and a lack of government support are only a few of the issues that farmers must deal with. Farmers and business owners claimed that this is preventing the sector from truly taking off [9]. To overcome these challenges and boost the production and trade of roses in Nepal, investing in agricultural research, providing training and support to farmers, improving infrastructure, and developing strategies to access international markets more effectively would be essential. Additionally, promoting the value-added aspects of the rose industry, such as rose-based products like perfumes or cosmetics, could also enhance its economic viability.

### 2.1 Floriculture sector help in ecosystem services, amelioration of climate

Floriculture encompasses the cultivation of a diverse array of flowering plants, from native to exotic species, thereby enhancing plant biodiversity within ecosystems and providing essential resources such as food and habitat for pollinators, birds, and other wildlife [19] This practice often involves the propagation and conservation of endangered or rare plant species, thereby preserving genetic diversity and contributing significantly to biodiversity conservation [20]. Advanced propagation techniques, such as tissue culture, micropropagation, and seed germination, are frequently employed in floriculture, particularly for endangered and threatened species. These techniques facilitate the rapid multiplication of plants, increasing their population size and genetic diversity, which supports future reintroduction or restoration efforts [21].

Moreover, floriculture plays a pivotal role in habitat creation, with greenhouses providing sheltered environments that support year-round plant growth and offer nesting sites and food for birds, insects, and small mammals [22].The cultivation of rare and endangered species in controlled environments, such as botanical gardens, nurseries, and specialized facilities, forms an integral part of "ex situ" conservation strategies. These environments offer a protected refuge for endangered plants, safeguarding them from threats in their natural habitats [23].

Additionally, floriculture enhances soil health through the incorporation of organic matter, such as compost and cover crops, which improves soil structure, increases nutrient availability, and boosts microbial activity [24]. Water conservation is another critical aspect, with techniques like drip irrigation, proper irrigation timing, mulching, and greenhouse design aimed at minimizing water usage [25]. Furthermore, floriculture contributes to climate regulation by sequestering carbon dioxide (CO2) through photosynthesis, particularly within greenhouse environments [26]. The Nepal rose trade offers numerous research opportunities due to several identified gaps in the existing literature. There is a lack of comprehensive market analyses, particularly regarding the structure, size, and dynamics of both local and international markets, as well as the value chain from production to retail. Economic studies are limited, especially those evaluating the trade's contribution to GDP, employment, and cost-benefit aspects. Modern farming techniques and productivity have not been extensively examined, nor have the impacts of trade policies and market access challenges on export competitiveness. Further research is needed on the environmental and social impacts, including sustainability practices and social dynamics within farming communities. Additionally, consumer behavior, technological integration, and the effects of climate change on rose production are underexplored areas. Comparative studies with leading rose-producing countries and evaluations of government initiatives and institutional support could provide valuable benchmarks and best practices.

## 3 Methodology

This case study was conducted to evaluate and understand the trade scenario of roses in Nepal over a ten-year period from 2013 to 2022. Data were sourced from government sites, specifically the Trade and Export Promotion Center (TEPC) and FAOSTAT. The obtained data were evaluated and presented in various forms of graphs for easy and timely understanding. Additionally, secondary materials such as interviews with florists (Kathmandu, Chitwan), articles from journals, newspapers, magazines, and other interviews were utilized. Relevant information was carefully selected to ensure thorough processing of the data.

## 4 Result and discussion

1 Imports of roses either cut flowers or whole plant
i As a cut flower

**Fig. 1:**
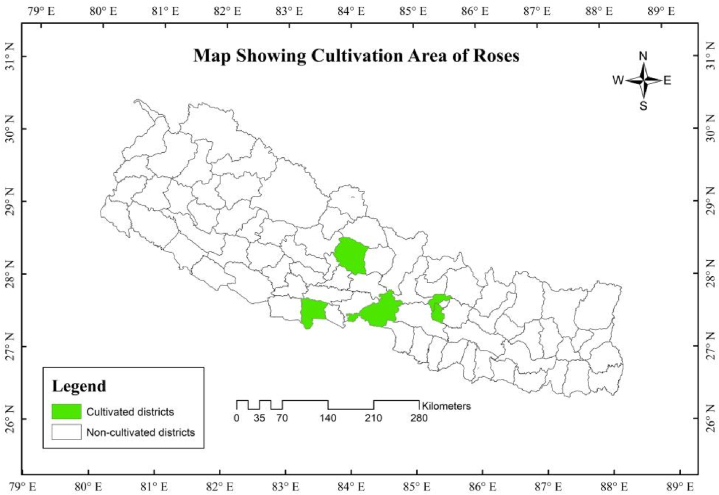
Administrative map of Nepal focusing on roses cultivation areas (4).

**Fig. 2:**
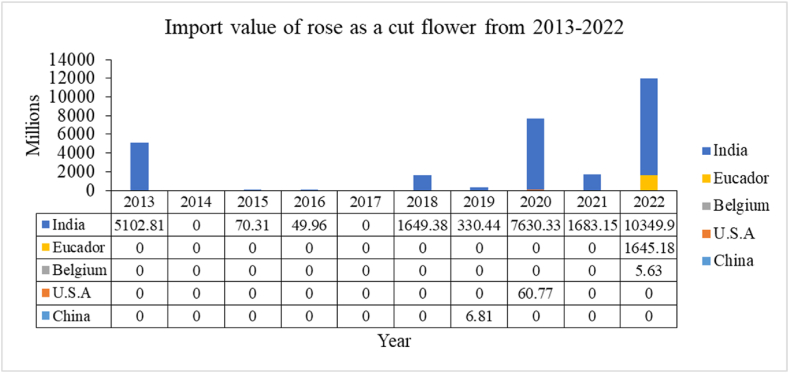
Import value of rose as a cut flower.

Examining the above data, Nepal has been importing rose as a cut flower from different nations like India, U.S.A, China, Ecuador, Belgium, etc (see Fig. 1). Although these nations are supplying a good amount of rose as a cut flower yearly the continuity in their supplement is not uniform. Nepal has not imported flowers from China since 2020. However, Belgium and Ecuador have become new suppliers to Nepal in recent years. India has consistently supplied the required amount of flowers to Nepal each year. (Fig. 2)[27]**.**

Source:Trade and export promotion center

The import from India has been regular and in higher volume due to the lower tariff rate of 6 %, compared to the 10 % tariff rate applied to other countries [27]. Due to the economic crisis caused by the pandemic, coupled with improper weather conditions for rose production in the country, the import of rose flowers increased drastically compared to previous years [28]. After 2020, Nepal stopped importing roses from China, but the import of roses from India continued steadily. This was due to flight restrictions caused by the COVID-19 pandemic [29].

ii. As grafted rose plants

**Fig. 3:**
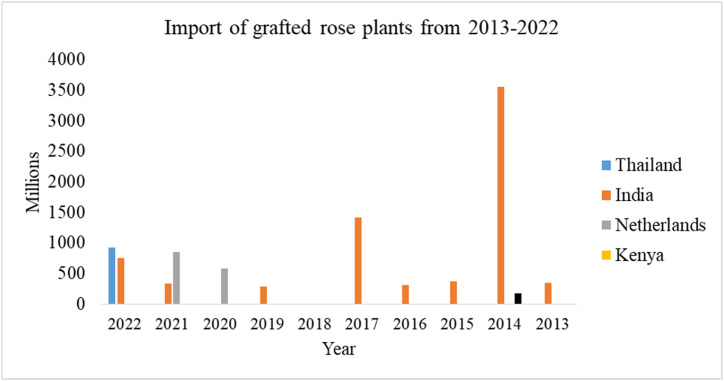
Import values of rose as grafted plants.

Fig. 3 shows Nepal imported grafted rose plants from different countries. Analyzing the graph India supplies grafted rose plants regularly with the highest supply of 3500$ in the year 2014. Supply from other countries is in piece ratio i.e. no other countries are regular suppliers along with India, Kenya or Netherlands or Thailand or few not specified countries are fulfilling the country demand for grafted rose plants. In the recent period, Nepal has stopped importing from African countries and is importing rose plants from European countries and India. Import in the year 2015 was low due to the occurrence of devastating earthquake in Nepal whereas during 2019–2020 it was low due to covid pandemic.

Source:Trade and export promotion center

The absence of proper tissue culture techniques forced Nepal to import quality plant materials from India in the past [30]. Dominance of hybrid varieties, cultivars with flowers having more attractive color and greater flower diameter, short height varieties as a result more plants can be accommodate in small piece of land.

iii. Monthly import value of rose as a cut flower

**Fig. 4:**
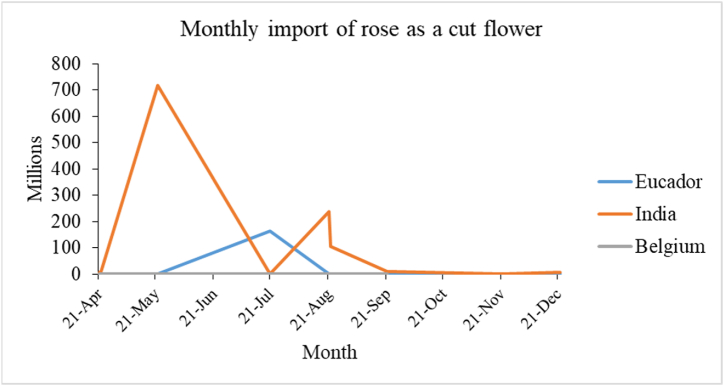
Monthly import value rose from 2021 April to 2022 December.

To know the monthly import trend of roses as a cut flower above graph was plotted. Analyzing the past data, from past India not only has been the top importer of rose yearly but also monthly. In the period of 2022 Feb–March Nepal imported rose from Belgium. Recently the import has decreased and Nepal started importing rose from Ecuador (the leading rose producer country in the world) ***[***Fig. 4***].***

Source:Trade and export promotion center

Due to COVID-19, big events and celebrations number were limited outcomes bringing less consumption of roses in Dec 2021–feb 2022 despite being the time of Valentine's [29]. Due to the government's decision to close markets and restrict transportation, the availability of rose to the general public supply of roses was limited, and those who do have them must pay a premium price, which reduces demand [28]. Rose is famous among youngsters but the shutdown of college during the Valentine's period was another reason for the low transaction of roses in Feb 2022 [29]. During April and May of 2022, the import of rose increased this was because of the high demand for a celebration of the country's new year 2079, the demand for rose as bouquet for the winner in the local election held the month of Baisakh 2079, the demand was also high due to the month of marriage in Nepal.

2 Export of roses either cut flower or whole plant

**Fig. 5:**
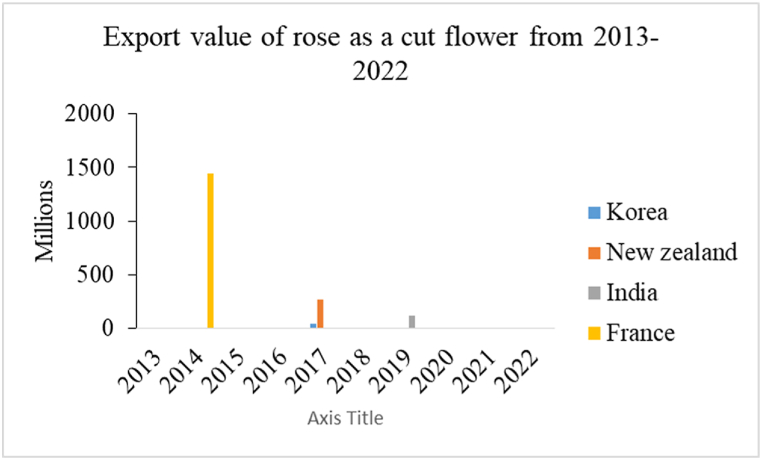
The export value of cut rose flower.

The above information illustrates export of roses is not satisfactory. Observing the past 10 years data the supply scenario of rose is quite imbalanced. In the past Nepal used to supply rose to countries like France, India, New Zealand, and Korea with the highest supply of 1500$ in the year 2014 to France. India is the regular importer of rose but the supply to India is seen only in a single year i.e. 2019 (during the period of covid 19) during the entire 10-year period. Since 2020 Nepal's rose market has had no supply to the international market ***[***Fig. 5***]*.**

Source:Trade and export promotion center

The Covid pandemic was the major potential reason for a great decrease in the supply of roses from Nepal as due to the pandemic investment in floriculture has been reduced [30]. Furthermore, the deterioration in the quality of produced roses was the reason behind the downfall in the supply of roses [30]. The farming families and the entrepreneurs involved in the floriculture business were reduced by 10 % this was also another factor that hindered the production of roses ultimately affecting the export process [31].

According to florists operating business in different parts of the country like Kathmandu, Butwal, Dhangadhi, and Chitwan, the demand rose in the country is fulfilled by the country production along with supply from India, Thailand, Ecuador, Netherlands, etc. But most of the trade of Nepal is with India. According to Pant Nursey and Flower Shop operating in Dhangadhi, the reason behind most trade with India might be geographic proximity, cultural and seasonal demand, established supply chains, competitive pricing, quality assurance, varietal availability, etc.

## 5 Conclusion

Agriculture being the backbone of development in Nepal, the progress of the floriculture sector can be regarded as negligible. Compared to other agriculture sectors, the floriculture branch has limited work and projects. Development of new varieties, new technologies, government subsidy schemes, transportation and storage facilities are not up to the satisfaction level. Research on the economic dynamics of the Nepal rose trade offers substantial practical benefits to various stakeholders. In-depth insights into market structure and dynamics can aid policymakers in developing informed trade policies and support programs, thereby enhancing the competitiveness of Nepalese roses. Understanding the economic contributions of the trade can underscore its role in GDP and employment, advocating for increased investment. Analyzing modern farming techniques can reveal best practices for increased yields and sustainability, while insights into trade policies can assist exporters in overcoming barriers and accessing new markets. Research on sustainable practices and the impacts of climate change can promote environmentally friendly methods, ensuring long-term viability. Exploring social dynamics can advocate for improved working conditions and gender equality. Consumer behavior studies can help tailor marketing strategies, and investigating digital platforms can streamline supply chains. Comparative studies with other countries can provide benchmarks and best practices, while evaluating institutional support can lead to the development of robust frameworks for sustainable growth. Overall, this research can drive significant improvements in policy, practice, and market strategies, fostering a thriving and sustainable rose trade in Nepal.

## CRediT authorship contribution statement

**Prakash Awasthi:** Writing – original draft, Visualization, Validation, Supervision, Resources, Methodology, Data curation, Conceptualization. **Shiva Prasad Adhikari:** Writing – review & editing.

## Declaration of competing interest

The authors declare that they have no known competing financial interests or personal relationships that could have appeared to influence the work reported in this paper.
